# Editorial: Insights in microbe and virus interactions with plants: 2022

**DOI:** 10.3389/fmicb.2023.1327245

**Published:** 2023-12-01

**Authors:** Ajar Nath Yadav, Tofazzal Islam

**Affiliations:** ^1^Department of Biotechnology, Dr. Khem Singh Gill Akal College of Agriculture, Eternal University, Baru Sahib, Himachal Pradesh, India; ^2^Department of Biotechnology, Faculty of Health and Life Sciences, INTI International University, Persiaran Perdana BBN Putra Nilai, Nilai, Negeri Sembilan, Malaysia; ^3^Institute of Biotechnology and Genetic Engineering (IBGE), Bangabandhu Sheikh Mujibur Rahman Agricultural University, Gazipur, Bangladesh

**Keywords:** biocontrol, microbial diversity, plant growth promotion, plant microbiome, sustainability

Plants are important mediators of interactions between their associated microbial communities. Plant microbial interaction is empirical research that represents a wide range of molecular interactions between pathogens and plants. Plant associated microbes can enhance stress resistance through the induction of several mechanisms. The interaction between microbiomes and plants is beneficial as well as pathogenic. These microorganisms have a significant impact on plant production and health in natural contexts and are capable of forming intricate co-associations with plants. The development of novel sequencing technology for examining plant microbiomes enables potential opportunities for depth analysis of plant interactions under various management strategies (Islam et al., 2023).

Over the past 450 million years, microbes and plants have associated with each other and formed an assemblage of species known as holobiont. Plants cohabit with different groups of microbes including bacteria, fungi, and archaea, which form complex microbial consortia. Microbe-plant associations in soil occur by the mean of chemical-based interactions (Hassani et al., 2018). Plants influence their interaction with the microbes and soil by releasing rhizodeposits comprised of diverse compounds such as root caps, border cells, volatile organic compounds, soluble exudates, and lost carbon. Among all rhizodeposits, released root exudated modulate the composition of microbes around the plant roots, as they act as chemo-attractants. The plant-microbe interaction varies due to differential gene expression in the microbes (Farrar et al., 2014). Both plants and microbes tend to benefit from each other, for example, plants provide novel metabolites that could be used by the microbes as an energy source. On the other hand, microbes guard the plants from various biotic and abiotic factors of the environment through two different mechanisms i.e., direct, and indirect. Plants' growth and development have been known to be affected by various biotic factors such as pests, insects, and pathogens such as pathogenic bacteria, fungi, and viruses (Yadav et al., 2023b). Through various indirect mechanisms such as the production of antibiotics, lytic enzymes, siderophores, and hydrogen cyanide, microbes lower the concentration of ethylene through the production of 1-aminocyclopropane-1-carboxylate (ACC) deaminase shields the plant health and suppresses the growth of pests and pathogens (Yadav et al., 2023a). A lack of nutrient supply and abiotic factors such as drought, salt, and high/low temperature have also been found to deplete the growth of the plants. Microbes alleviate this stress via direct plant growth mechanisms (Devi et al., 2022). Microbes alleviate the nutrients and abiotic stress by availing the soluble form through mechanisms such as nitrogen fixation, nutrients (zinc, potassium, and phosphorus) solubilization, siderophores production for the chelation of iron, ACC deaminase activity, activation of antioxidants, and the production of various phytohormones such as auxins, cytokinin and gibberellins (Kour et al., 2023; Islam et al., 2023).

This Research Topic includes high-quality research papers and review articles focused on new insights, novel developments, current challenges, latest discoveries, recent advances, and future perspectives in the field. The Research Topic contains six research and three review papers that cover different aspects of viral, bacterial, and fungal pathogens in relation to plant-microbe molecular interaction, plant colonization, detection, and disease control. Canfora et al. evaluated the impact of strains of Beauveria brongniartii and Beauveria bassiana on soil bacterial and fungal communities using an approach based on the terminal restriction fragment polymorphism (T-RFLP) analysis. The findings conclude that the application of the bioinocula induced only a transient and limited effect on the soil microbial community; even though some changes in the structure dynamic and frequency of soil bacterial and fungal OTUs emerged.

Cui et al. isolated and characterized *Priestia megaterium* KD7 for the biological control of pear fire blight. Fire blight, caused by the bacterium *Erwinia amylovora*, is one of the most serious plant diseases that mainly affects Rosaceae, such as apple, pear, medlar, and quince. Biological control with microbial antagonists is considered a powerful and eco-friendly alternative to controlling fire blight. The findings indicate that *P. megaterium* KD7 could be used as a potential source of a new biocontrol agent to control fire blight.

In another article, Xi et al. evaluated the microbiome diversity, composition, and assembly in a *California citrus* orchard. The environmental impact of agrochemical pesticides and fertilizers leads to changes in consumer behavior toward sustainably grown food and food products and as a result, farmers are increasingly relying on biological-based technologies and less on synthetic chemistries. Microbiomes have been shown to provide many benefits to plants by priming the immune system and protecting them from diseases, facilitating nutrient acquisition, and overall enhancing health and increasing yield. Data indicated that compartmentalization of microbiomes with distinct profiles occurs between above and below ground microbial communities. These findings highlight key microbial taxa that could be engineered as biopesticides and biofertilizers for citriculture. Interaction between the flagellum of *Candidatus* Liberibacter asiaticus and the vitellogenin-like protein of *Diaphorina citri* significantly influences *C*Las titer evaluated by Peng et al.. As a regulatory factor, *Vg_VWD* was upregulated in *C*Las-infected *D. citri* compared with uninfected ones, and the *C*Las titer increased significantly after *Vg_VWD* was silenced. The study provides a foundation for studying the roles that flaA and *Vg_VWD* may play together or separately in insect and plant hosts.

Deja-Sikora et al. explored the potential role of *Rhizophagus irregularis* and *Funneliformis mosseae* for the growth of *Solanum tuberosum* L. in a different way. This study indicated that multipartite interactions can take place in plant hosts inhabited by phytopathogens and endophytes. The application of AMF inoculum can reduce the economic losses caused by the virus. In the study of Duduk et al. the correlation between the occurrence of sugar beet RTD and the presence of root rot fungal pathogens in a semi-field “*Ca*. P. solani” transmission experiment with the cixiid vector *Reptalus quinquecostatus* (Dufour), in addition to naturally infected sugar beet in the open field. Zboralski and Filion summarized the effects of climate change-induced stresses on plants and detailed the mechanisms used by plant-beneficial *Pseudomonas* strains to alleviate them. Recommendations are made to promote targeted research on the stress-alleviating potential of these bacteria.

Another review by Kumar et al. on *Stenotrophomonas* in diversified cropping systems. The review discusses various plant growth and biocontrol attributes of the genus *Stenotrophomonas* in various food crops along with knowledge gaps. Additionally, the potential risks and challenges associated with the use of *Stenotrophomonas* in agriculture systems have also been discussed along with a call for further research in this area.

Al-Turki et al. summarize recent advances in PGPR-mediated resilience toward interactive effects of drought and salt stress in plants. The advancements made in the field of PGPR-mediated resilience through multi-omics approaches (*viz*., genomics, transcriptomics, proteomics, and metabolomics) to unravel the intricate interactions between PGPR and plants have been discussed, including the molecular pathways involved in stress tolerance.

Research on plant microbes has greatly increased in the past few years. Integrated strategies such as multi-omics, engineering, theory, experimental biology, computational biology, and statistics offer quantitative insights into plant microbiome interactions (Islam et al., 2023). The importance of positive interactions between viruses and microbes in the plant has frequently been overlooked in plant breeding programmes, which have traditionally focused on examining the genetic variability of the crop for improved yield and stress tolerance. In the future, these integrated strategies will offer methods for evaluating as well as implementing the use of plant microbiome interaction to raise the productivity and sustainability of global agriculture.

## Author contributions

AY: Writing – original draft. TI: Writing – review & editing.
